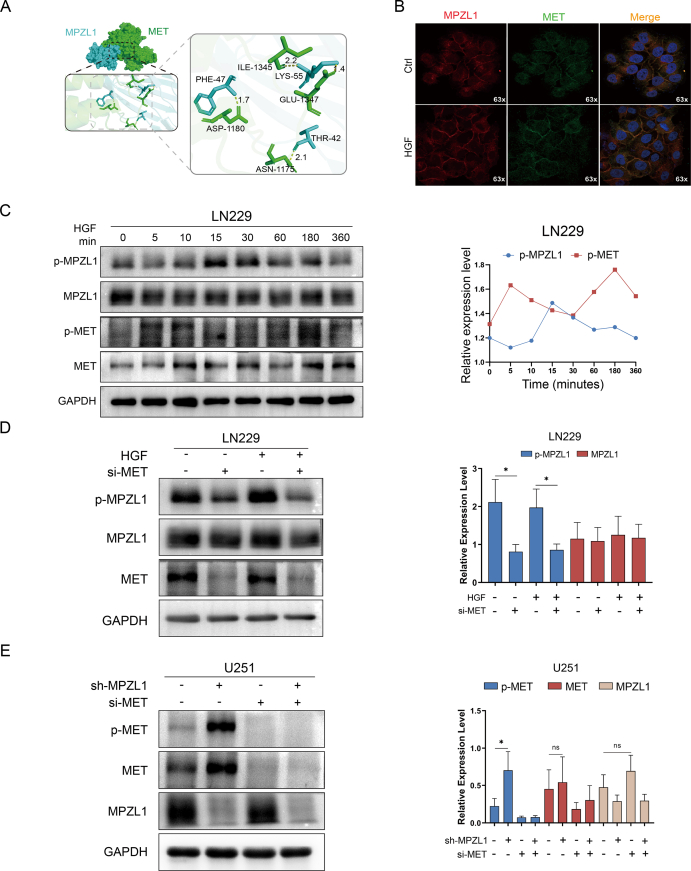# MPZL1 as an HGF/MET signaling amplifier promotes cell migration and invasion in glioblastoma

**DOI:** 10.1016/j.gendis.2023.101085

**Published:** 2023-09-09

**Authors:** Haimin Song, Bowen Ni, Xuetao Peng, Weijuan Xu, Shaochun Yang, Runwei Yang, Ziyu Wang, Kaishu Li, Rui Lin, Yunxiao Zhang, Jinglin Guo, Kezhi Wu, Guangwei Shi, Jichao Sun, Chunming He, Yawei Liu

**Affiliations:** a Department of Neurosurgery, The First Affiliated Hospital of Gannan Medical University, Ganzhou, Jiangxi 341000, China; b Department of Neurosurgery & Medical Research Center, Shunde Hospital, Southern Medical University (The First People's Hospital of Shunde Foshan), Foshan, Guangdong 528300, China; c Medical Research Center, Shunde Hospital, Southern Medical University (The First People's Hospital of Shunde Foshan), Foshan, Guangdong 528300, China; d Bidding and Procurement Office, Shunde Hospital, Southern Medical University (The First People's Hospital of Shunde Foshan), Foshan, Guangdong 528300, China; e Department of Neurosurgery, Nanfang Hospital, Southern Medical University, Guangzhou, Guangdong 510515, China; f Department of Neurosurgery, The Sixth Affiliated Hospital of Guangzhou Medical University, Qingyuan, Guangdong 511518, China; g Department of Neurosurgery, The First Affiliated Hospital of Shantou University Medical College, Shantou, Guangdong 515041, China; h Department of Geriatrics, Shenzhen People's Hospital (The Second Clinical Medical College, Jinan University; The First Affiliated Hospital, Southern University of Science and Technology), Shenzhen, Guangdong 518020, China

The extremely poor prognosis of patients is largely due to hepatocyte growth factor (HGF)/MET signaling, which promotes migration and invasion of glioblastoma (IDH wild-type; GBM; WHO grade 4).^1^^,^^2^ Clinical trials targeting MET, the only receptor of HGF, have yielded unimpressive results in GBM.^3^^,^^4^ Here we found that HGF induced strong chemotaxis on GBM cells, but MET expression was extremely low. We, therefore, used single-cell RNA sequencing (scRNA-seq) coupled with label-free proteome profiling to identify membrane proteins associated with HGF/MET signaling amplification in GBM and to provide a novel modulator, MPZL1, for HGF/MET-targeted therapy.

HGF, epidermal growth factor (EGF), and fibroblast growth factor (FGF) can induce GBM cell migration and invasion. Analysis of scRNA-seq data from clinical GBM samples and CGGA and GEO databases showed that HGF expression was the highest and distributed across a broad range of cell types (Fig. 1A and B; Fig. S1A–C). C6 and U87 cells were stimulated with these cytokines at different concentrations followed by transwell assays. The results showed that HGF at a very low concentration (0.1 nM) had the strongest chemotactic effect compared with EGF (10 nM) and FGF (50 nM) (Fig. 1C; Fig. S1D–K). Bulk RNA-seq data from TCGA, GTEx, and GEO databases were used to analyze MET expression in GBM tissues and normal brain tissues, as MET is the only receptor for HGF. The results showed that MET expression in GBM tissues was not significantly different from that in normal brain tissues (Fig. 1D). MET expression was higher in normal brain tissues compared with low-grade gliomas or pan-gliomas (Fig. 1D; Fig. S1L, M). Notably, MET expression was extremely low in glioma tissues and was barely detectable by Western blot (Fig. S1N). These results suggest the existence of another underlying mechanism that plays a critical role in HGF/MET signaling.

**Figure 1:**
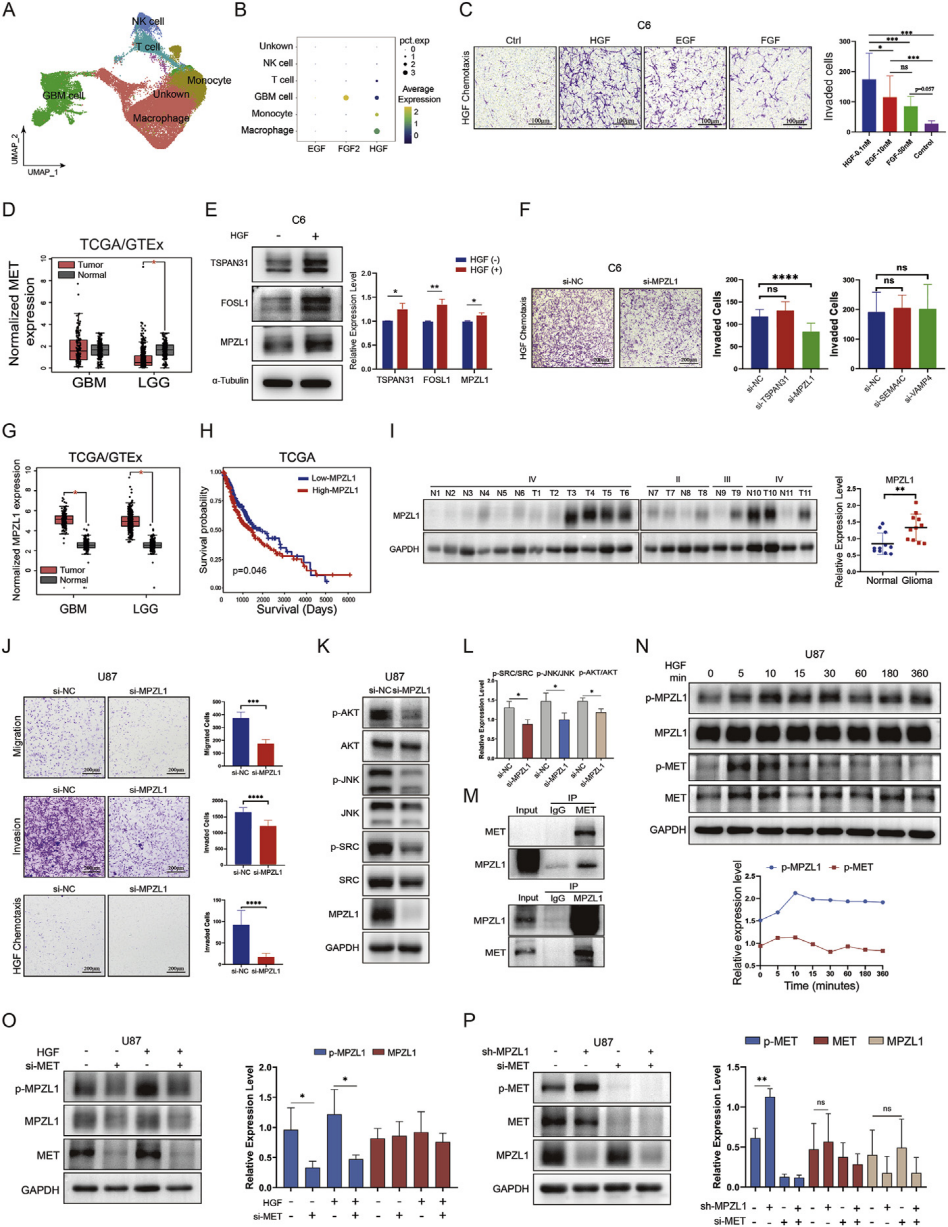
MPZL1 amplifies HGF/MET signaling and promotes cell migration and invasion in glioblastoma. **(A)** The UMAP plots showing distinct clusters of cells derived from scRNA-seq data of clinical GBM tissues. **(B)** Dot plots of HGF, EGF, and FGF2 expression among different types of cells. **(C)** Chemotaxis of C6 cells by HGF, EGF, and FGF shown by transwell invasion assays. Quantitative histograms show the motility of C6 cells in transwell invasion assays at concentrations where HGF, EGF, and FGF each exert their maximal chemotaxis. **(D)** The box plot showing the mRNA expression level of MET between TCGA glioma samples (GBM and LGG) and GTEx normal brain tissues. **(E)** Changes in TSPAN31, FOSL1, and MPZL1 protein expression in C6 cells upon HGF stimulation verified by Western blot. **(F)** The results of the transwell invasion assay showed that the knockdown of MPZL1 affected the invasive ability of C6 cells. Quantitative histograms show the changes in the invasive ability of C6 cells after the knockdown of Tspan31, Vamp4, Sema4c, or Mpzl1 as analyzed by transwell invasion assays. **(G)** The box plot showing the mRNA expression level of MPZL1 between TCGA glioma samples (GBM and LGG) and GTEx normal brain tissues. **(H)** Kaplan-Meier survival curves for glioma patients stratified by MPZL1 expression levels based on the clinical information from the TCGA database. **(I)** Scatter statistics of MPZL1 expression in glioma tissues and normal brain tissues detected by Western blot. **(J)** Effect of MPZL1 knockdown on U87 cell migration and invasion in the absence of HGF stimulation and effect of MPZL1 knockdown on U87 cell invasion under HGF stimulation. **(K)** Changes in phosphorylation levels of AKT, JNK, and SRC following knockdown of MPZL1 in U87 cells detected by Western blot. **(L)** The quantitative histograms showing the changes in phosphorylation levels of AKT, JNK, and SRC following MPZL1 knockdown in U87 cells detected by Western blot. **(M)** Protein interactions between MET and MPZL1 validated by Co-IP experiments. **(N)** Changes in MET and MPZL1 phosphorylation levels upon HGF stimulation lasting for different periods in U87 cells detected by Western blot and the statistical line chart. **(O)** Effect of MET knockdown with or without HGF stimulation on MPZL1 protein expression and MPZL1 phosphorylation levels evaluated by Western blot and quantitative histograms. **(P)** Effect of MPZL1 knockdown with or without HGF stimulation on MET protein expression and MET phosphorylation levels evaluated by Western blot and quantitative histograms. ns, not significant; ^∗^*P* < 0.05, ^∗∗^*P* < 0.01, ^∗∗∗^*P* < 0.001, ^∗∗∗∗^*P* < 0.0001.

All C6 cells in the Boyden chamber were divided into the HGF chemotaxis induction group (HGF (+)) and control group (HGF (−)) and were collected for label-free proteome profiling (Fig. S2A). The screening conditions for differentially expressed proteins between the two groups were a fold-change >2-fold and a *P*-value < 0.05. A total of 67 differentially expressed proteins were obtained (Fig. S2B). GO (Gene Ontology) enrichment analysis of the differentially expressed proteins showed that pathways such as “positive regulation of cell cycle” and “mitotic cytokinesis” were up-regulated in the HGF (+) group (Fig. S2C). The results of the proteomic analysis were verified by Western blot, which showed that tetraspanin-31 (TSPAN31), fos-related antigen 1 (FOSL1), and myelin protein zero-like protein 1 (MPZL1) were all significantly up-regulated in HGF-stimulated cells (Fig. 1E).

To screen for proteins that play a key role in HGF chemotaxis to GBM cells, the list of differentially expressed proteins was analyzed and four proteins were selected based on fold change and their correlation with cell motility. These four proteins were vesicle-associated membrane protein 4 (VAMP4), TSPAN31, MPZL1, and semaphorin-4C (SEMA4C). To further confirm the effect of these candidate proteins on GBM cell invasion under HGF stimulation, three small interfering RNA (siRNA) sequences were designed to knock them down (Fig. S2D). The transwell invasion assay under HGF stimulation showed that the invasive ability of C6 cells was significantly reduced only when MPZL1 was knocked down (Fig. 1F). These results suggest that MPZL1 is a critical regulator of HGF-induced cell invasion. Therefore, we investigated MPZL1 in this study.

Figure 1G, S3A and S3B show that MPZL1 is expressed at higher levels in GBM compared with low-grade glioma and normal brain tissue (one-way ANOVA, *P* < 0.05). Further survival analysis showed that high MPZL1 expression was strongly associated with a poor prognosis in glioma patients (Fig. 1H; Fig. S3C–E; Kaplan Meier, *P* < 0.05). Moreover, Western blotting assays showed that the amount of MPZL1 protein was significantly up-regulated in glioma tissues compared with the normal (Fig. 1I; one-way ANOVA, *P* < 0.01).

The protein expression levels of MPZL1 were examined in commonly used GBM cell lines to select appropriate human-derived GBM cell lines (Fig. S4A, B). Transwell assays confirmed that the knockdown of MPZL1 in U87 and C6 cells significantly inhibited cell migration and invasion (Fig. 1J; Fig. S4C–G). Under HGF stimulation, MPZL1 knockdown was found to significantly attenuate the invasive ability (Fig. 1J; Fig. S4H). Furthermore, overexpression of MPZL1 significantly increased the migration and invasion of U118 and U251 cells (Fig. S4I–O). These results indicate that MPZL1 can promote the invasion or migration of GBM cells either in the presence or absence of HGF.

To explore the mechanism by which MPZL1 promotes cell invasion and migration, bulk RNA-seq data of glioma samples from the CGGA (693 & 325) database were ranked according to the expression level of MPZL1, and the median was used as the cut-off point to divide the samples into the high-MPZL1 group and the low-MPZL1 group. Differentially expressed genes between the two groups were selected by absolute fold-change > 2 with *P* < 0.05 as the threshold (Table S1), and GO and KEGG pathway enrichment analyses were performed (Fig. S5A–D). Further GSEA enrichment analysis showed that “focal adhesion” and “PI3K-Akt signaling pathway” were significantly up-regulated in the high MPZL1 expression group (Fig. S5E, F). MPZL1 has been reported to bind to the SRC homology domain phosphatase-2 (SHP-2). Detection of protein phosphorylation activation levels downstream of the SRC signaling pathway by Western blot revealed that MPZL1 knockdown significantly down-regulated protein phosphorylation levels of AKT, JNK, and SRC in U87, while MPZL1 overexpression significantly up-regulated them in U251 cells (Fig. 1K, L; Fig. S5G).

To further explore the association between MPZL1 and MET, scRNA-seq analysis showed that the expression level of MPZL1 and the proportion of expressing cells were significantly higher than MET (Fig. S5H–K). A potential reciprocal binding between MPZL1 and MET was predicted by molecular docking^5^ and demonstrated by co-immunoprecipitation (co-IP) and immunofluorescence (IF) co-localization assay (Fig. 1M; Fig. S6A, B). MPZL1 co-localized with MET on the cell membrane of HELA cells, and HGF stimulation increased the co-localization signal (Fig. S6B).

Phosphorylation of MPZL1 and MET in U87 and LN229 cells did not occur simultaneously but sequentially upon sustained stimulation with HGF, as detected by Western blot. That is, MET phosphorylation levels peaked at 5–10 min after stimulation, whereas MPZL1 phosphorylation peaked at 10–30 min (Fig. 1N; Fig. S6C). These results suggest that the phosphorylation activation of MET preceded that of MPZL1 after HGF stimulation, and we hypothesized that the phosphorylation of MPZL1 was driven by MET. Therefore, MET was knocked down in U87 and LN229 cells, and it was found that this could lead to a significant decrease in the phosphorylation level of MPZL1 (Fig. 1O; Fig. S6D). However, there was no significant change in the total amount of protein for MPZL1, suggesting that MPZL1 expression is not regulated by MET. However, the phosphorylation level of MET was significantly increased after MPZL1 knockdown (Fig. 1P; Fig. S6E). This up-regulation is feedback regulated by the loss of MPZL1.

Taken together, MPZL1 may recruit downstream SRC to amplify the HGF/MET signaling and further promote GBM cell migration and invasion. This study reveals the relationship between MPZL1 and MET in the highly infiltrative and aggressive growth of GBM, providing theoretical support for the treatment based on targeting MPZL1.

## Ethics declaration

This study was approved by the Ethics Committee of Nanfang Hospital, Southern Medical University. Written informed consent was provided by all patients.

## Author contributions

YL and HS designed the study. HS, BN, SY, RY, ZW, and KL conducted various experiments and acquired and analyzed data. XP, WX, RL, YZ, JG, KW, and GS provided important reagents and valuable feedback on the manuscript. YL, BN, and CH wrote and edited the manuscript. JS contributed to the quality control of the manuscript. All authors read and approved the final manuscript.

## Conflict of interests

The authors declare that they have no competing interests.

## Funding

This study was supported by the 10.13039/501100003453Natural Science Foundation of Guangdong Province, China (No. 2022A1515012552), 10.13039/501100004791Shenzhen Science and Technology Innovation Committee of China (SZSTI; No. JCYJ20220818102611025), Research Initiation Project of Shunde Hospital, Southern Medical University (No. CRSP2022002), Research Initiation Project of the First Affiliated Hospital of Gannan Medical University (No. QD202316), and Beijing Sisco Clinical Oncology Research Foundation of China (No. Y-2022METAZMS-0118).

## Data availability

The scRNA-seq data used in this study are available at the Genome Sequence Archive (https://ngdc.cncb.ac.cn/gsa/) with the accession number CRA002498.

## Figures and Tables

**figs1:**
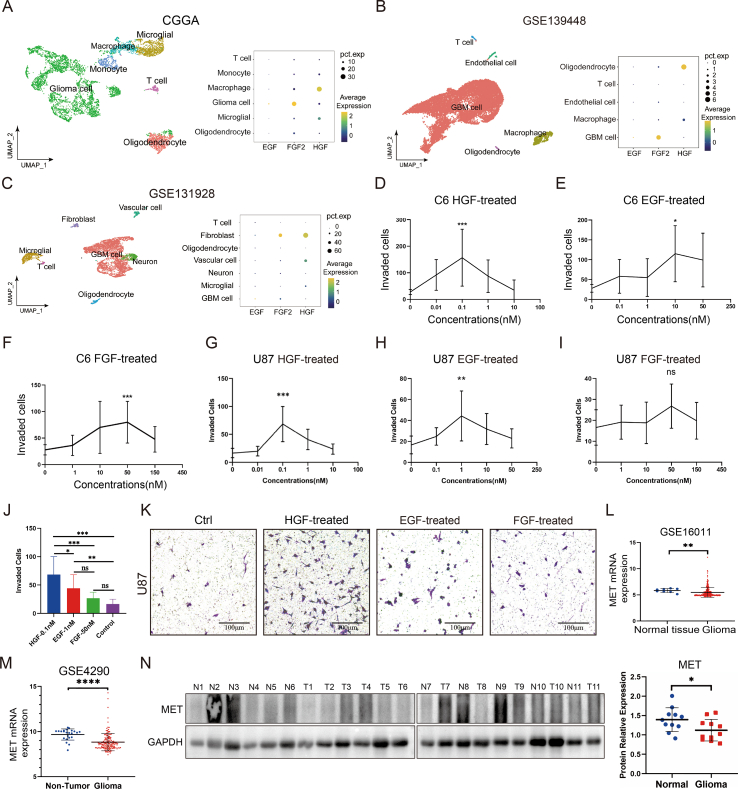


**figs2:**
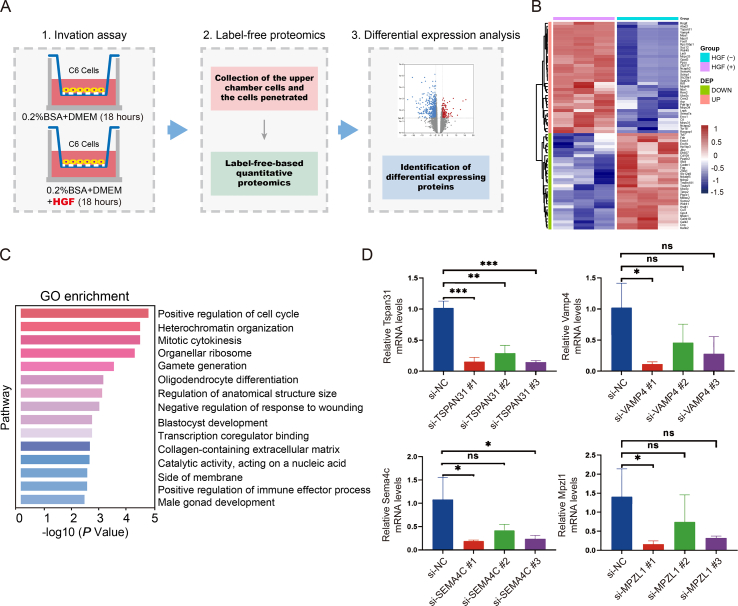


**figs3:**
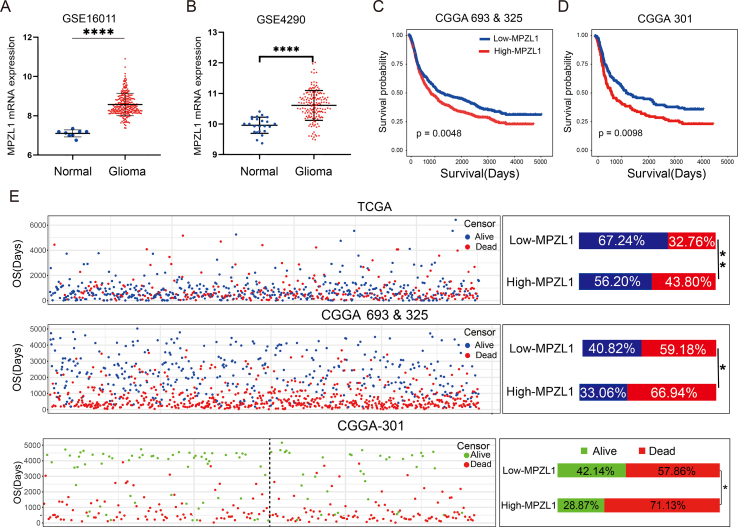


**figs4:**
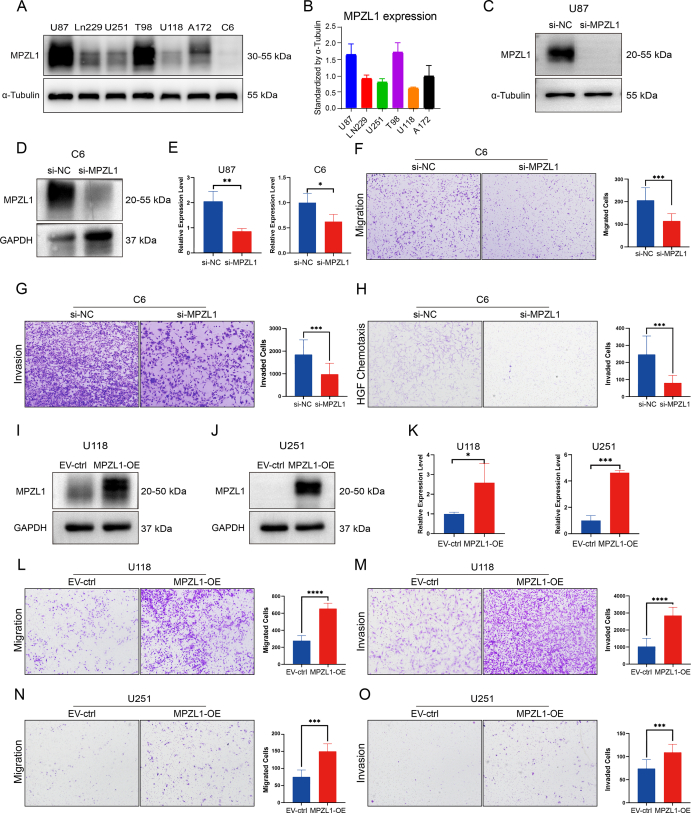


**figs5:**
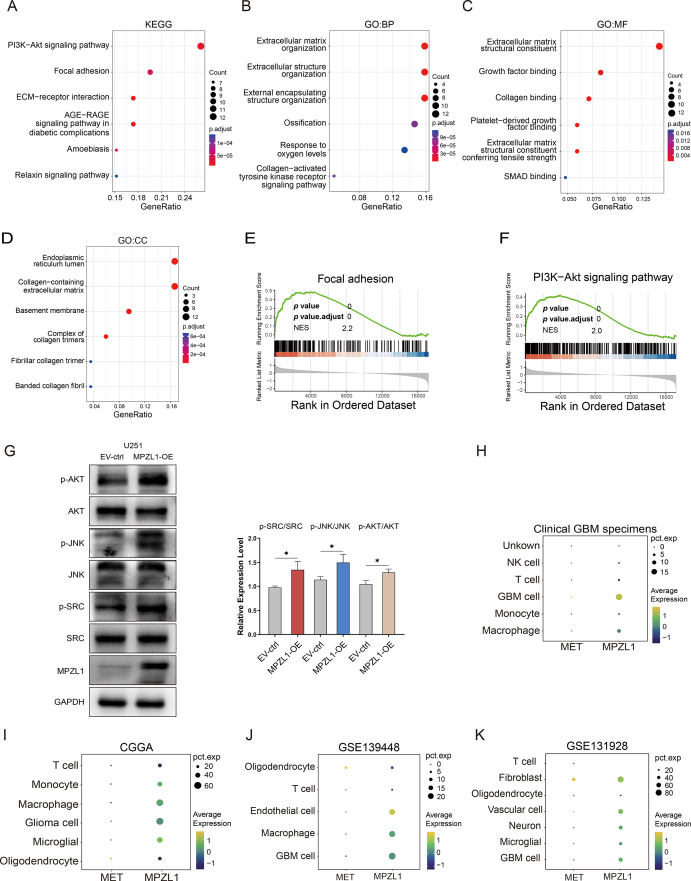


**figs6:**