# Supplementary material for: MPZL1 as an HGF/MET signaling amplifier promotes cell migration and invasion in glioblastoma
Source: Genes Dis. 2023 Sep 9;11(5):101085. doi: 10.1016/j.gendis.2023.101085 (PMC11176650; doi:10.1016/j.gendis.2023.101085)
Supplement: Multimedia component 1 [file mmc1.docx]

**Figure S1** Expression of HGF and MET in glioma and chemotaxis of HGF on GBM cells. **(A–C)** The UMAP plots showing the distinct clusters of cells derived from CGGA, GSE139448, and GSE131928 of the GEO database and the dot plots of HGF, EGF, and FGF2 expression levels among different types of cells. **(D–I)** The line graphs showing the motility of C6 and U87 cells in transwell invasion assays stimulated with different concentrations of HGF, EGF, and FGF. **(J)** The quantitative histograms showing the motility of U87 cells in transwell invasion assays at concentrations where HGF, EGF, and FGF each exert their maximal chemotaxis. **(K)** Chemotaxis of U87 cells by HGF, EGF, and FGF detected by transwell invasion assays. **(L)** The scatterplot showing the mRNA expression level of MET between glioma samples and normal brain tissues based on GSE16011 from the GEO database. **(M)** The scatterplot showing the mRNA expression level of MET between glioma samples and normal brain tissues based on GSE4290 from the GEO database. **(N)** Scatter statistics of MET expression in glioma tissues and normal brain tissues detected by Western blot. The bands of GAPDH shown here were performed in the same batch as the experiment in Figure 1I, and are therefore identical. ns, not significant; ^*^*P* < 0.05, ^**^*P* < 0.01, ^***^*P* < 0.001, ^****^*P* < 0.0001.

**Figure S2** Proteomic analysis and experimental validation. **(A)** Schematic illustration of comprehensive strategies for identifying the target protein related to HGF stimulation. **(B)** Heatmap of differentially expressed proteins between HGF (+) and HGF (−) cells. **(C)** GO enrichment analysis of differentially expressed proteins between HGF (+) and HGF (−) cells. **(D)** The quantitative histograms showing the knockdown of different siRNA sequences against TSPAN31, VAMP4, SEMA4C, and MPZL1 at the mRNA level verified by Q-PCR experiments. ns, not significant; ^*^*P* < 0.05, ^**^*P* < 0.01, ^***^*P* < 0.001.

**Figure S3** Expression of MPZL1 in glioma and its effect on the prognosis of glioma patients. **(A, B)** The scatterplot showing the mRNA expression level of MPZL1 between glioma samples and normal brain tissues based on GEO datasets. **(C, D)** Kaplan-Meier survival curves for glioma patients stratified by MPZL1 expression levels based on the clinical information from the CGGA database. **(E)** Survival analysis of glioma patients stratified by MPZL1 expression level based on clinical information from the TCGA and CGGA databases. ^*^*P* < 0.05, ^**^*P* < 0.01, ^****^*P* < 0.0001.

**Figure S4** Regulation of MPZL1 on HGF-mediated chemotactic effect on GBM cells. **(A)** Protein expression levels of MPZL1 in different GBM cell lines detected by Western blot. **(B)** Quantitative histogram of protein expression levels of MPZL1 in different GBM cell lines detected by Western blot. **(C, D)** The knockdown of MPZL1 protein expression in U87 and C6 cells detected by Western blot. **(E)** Quantitative histogram of Western blot assay for knockdown of MPZL1 protein expression in U87 and C6 cells. **(F)** Effect of MPZL1 knockdown on C6 cell migration in the absence of HGF stimulation. **(G)** Effect of MPZL1 knockdown on C6 cell invasion in the absence of HGF stimulation. **(H)** Effect of MPZL1 knockdown on C6 cell invasion under HGF stimulation. **(I, J)** The overexpression of MPZL1 protein expression in U118 and U251 cells detected by Western blot. **(K)** Quantitative histogram of Western blot assay for overexpression of MPZL1 protein expression in U118 and U251 cells. **(L)** Effect of MPZL1 overexpression on U118 cell migration in the absence of HGF stimulation. **(M)** Effect of MPZL1 overexpression on U118 cell invasion in the absence of HGF stimulation. **(N)** Effect of MPZL1 overexpression on U251 cell migration in the absence of HGF stimulation. **(O)** Effect of MPZL1 overexpression on U251 cell invasion in the absence of HGF stimulation. ^*^*P* < 0.05, ^**^*P* < 0.01, ^***^*P* < 0.001, ^****^*P* < 0.0001.

**Figure S5** Effect of MPZL1 on intracellular signaling pathways in GBM cells. **(A)** KEGG pathway enrichment analysis of differential genes between the high and low MPZL1 expression groups based on CGGA database GBM samples. **(B)** GO-BP enrichment analysis of differential genes between the high and low MPZL1 expression groups based on CGGA database GBM samples. **(C)** GO-MF enrichment analysis of differential genes between the high and low MPZL1 expression groups based on CGGA database GBM samples. **(D)** GO-CC enrichment analysis of differential genes between the high and low MPZL1 expression groups based on CGGA database GBM samples. **(E, F)** GSEA analysis of the signaling pathways up-regulated in the high MPZL1 expression group based on the CGGA-GBM dataset. **(G)** The quantitative histograms showing the changes in phosphorylation levels of AKT, JNK and SRC following MPZL1 overexpression in U251 cells detected by Western blot. **(H–K)** Dot plots of MPZL1 and MET expression levels among different types of cells based on scRNA-seq data from clinical GBM tissues, CGGA database, and GSE139448 and GSE131928 of GEO database. ^*^*P* < 0.05.

**Figure S6** Inter-regulatory relationship between MET and MPZL1. **(A)** Phyre2 predicted the three-dimensional structures of MET and MPZL1 proteins, performed molecular docking, and plotted the interacting amino acid sites with the PyMOL Molecular Graphics System (version 2.0 Schrodinger, LCC). **(B)** The co-localization of MET and MPZL1 in HELA cell membranes with or without HGF stimulation shown by immunofluorescence assays. **(C)** The changes in MET and MPZL1 phosphorylation levels upon HGF stimulation lasting for different periods in LN229 cells detected by Western blot and the statistical line chart. **(D)** The effect of MET knockdown with or without HGF stimulation on MPZL1 protein expression and MPZL1 phosphorylation levels evaluated by Western blot and the quantitative histograms. **(E)** The effect of MPZL1 knockdown with or without HGF stimulation on MET protein expression and MET phosphorylation levels evaluated by Western blot and the quantitative histograms. ns, not significant; ^*^*P* < 0.05.

**Tables**

**Table S1** Differentially expressed genes between the high-MPZL1 and low-MPZL1 groups.

**Table S2** Differentially expressed proteins of proteomics analysis.

**Table S3** Primers and siRNA sequences.

**Figure 1**


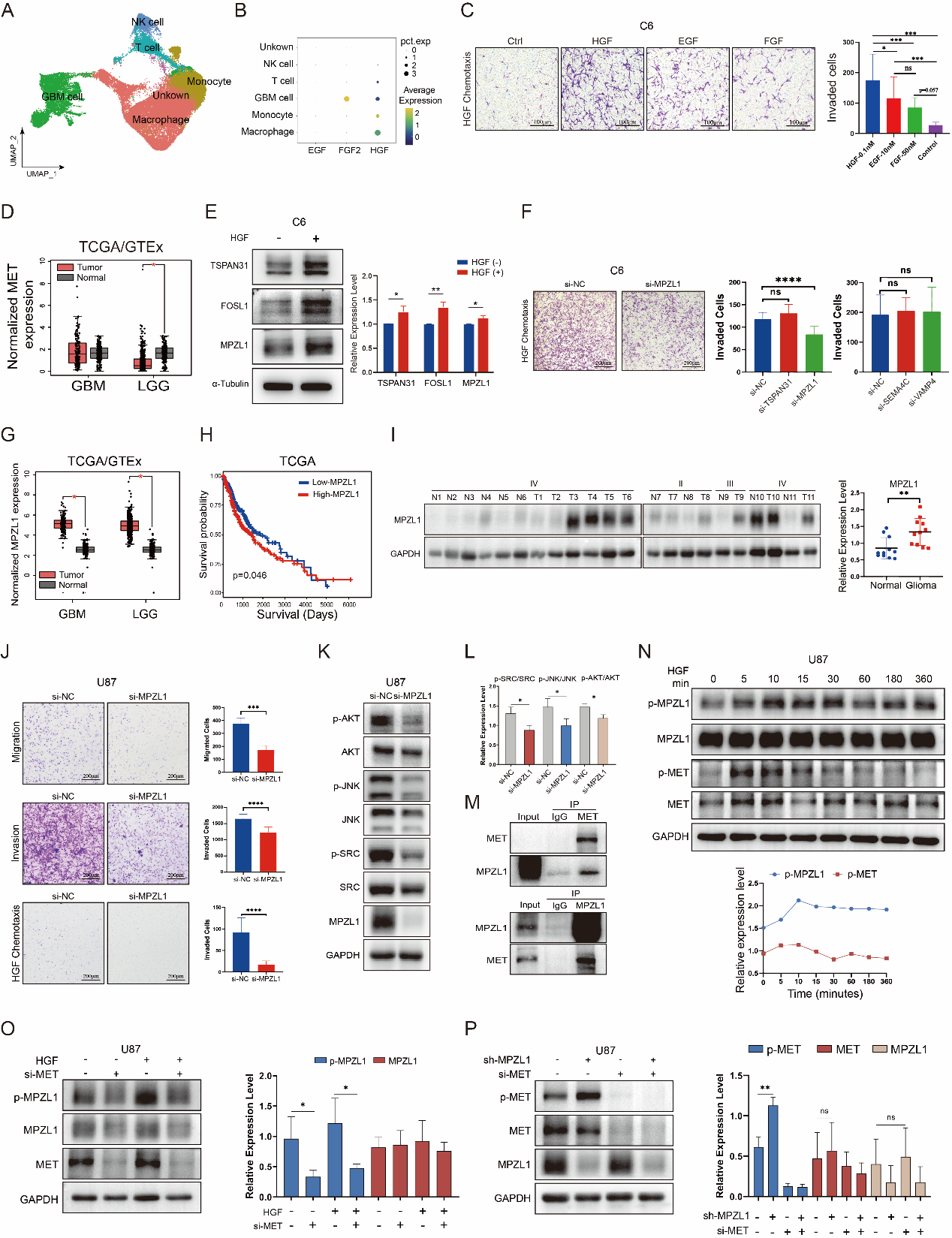


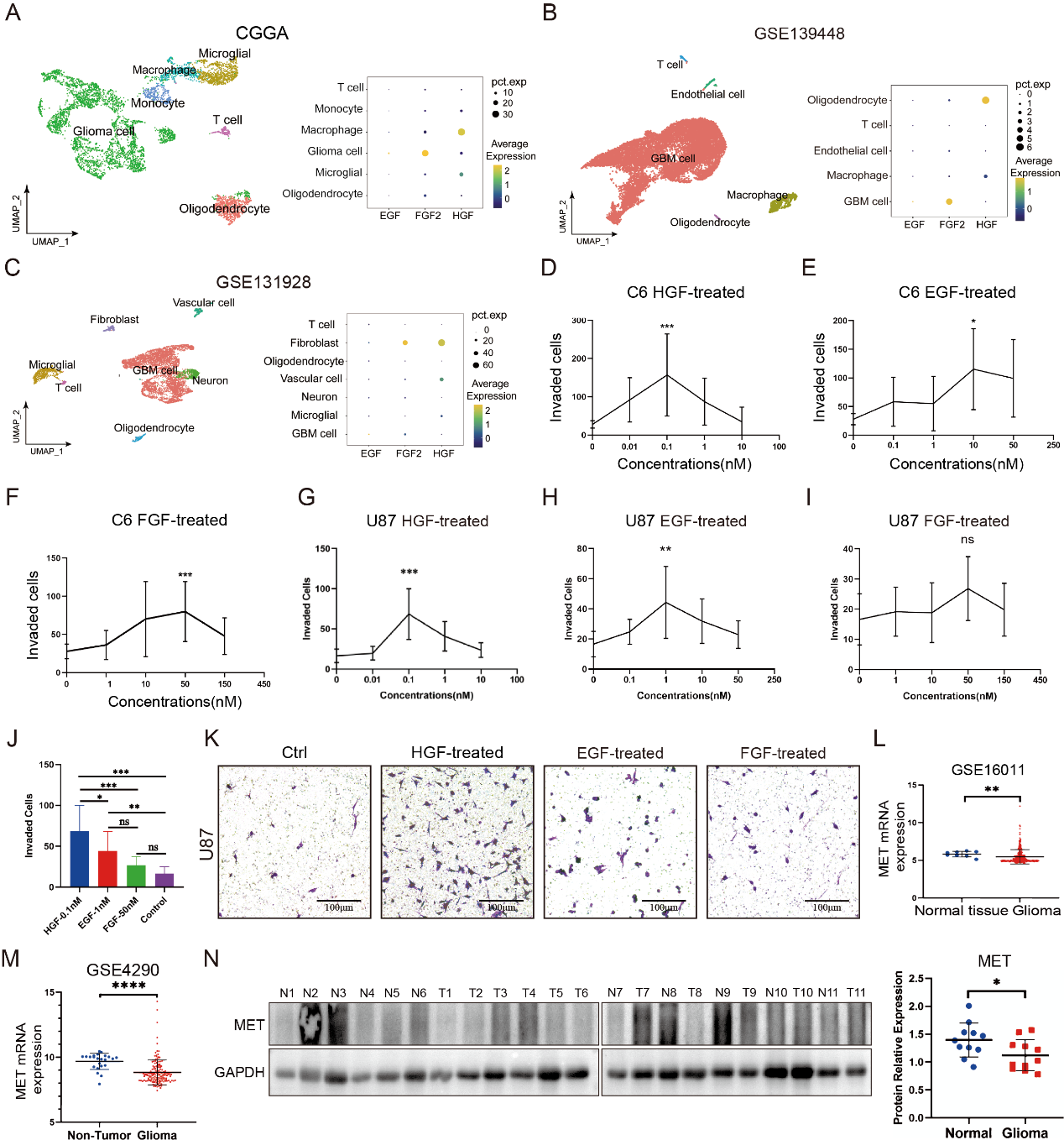
**Figure S1**


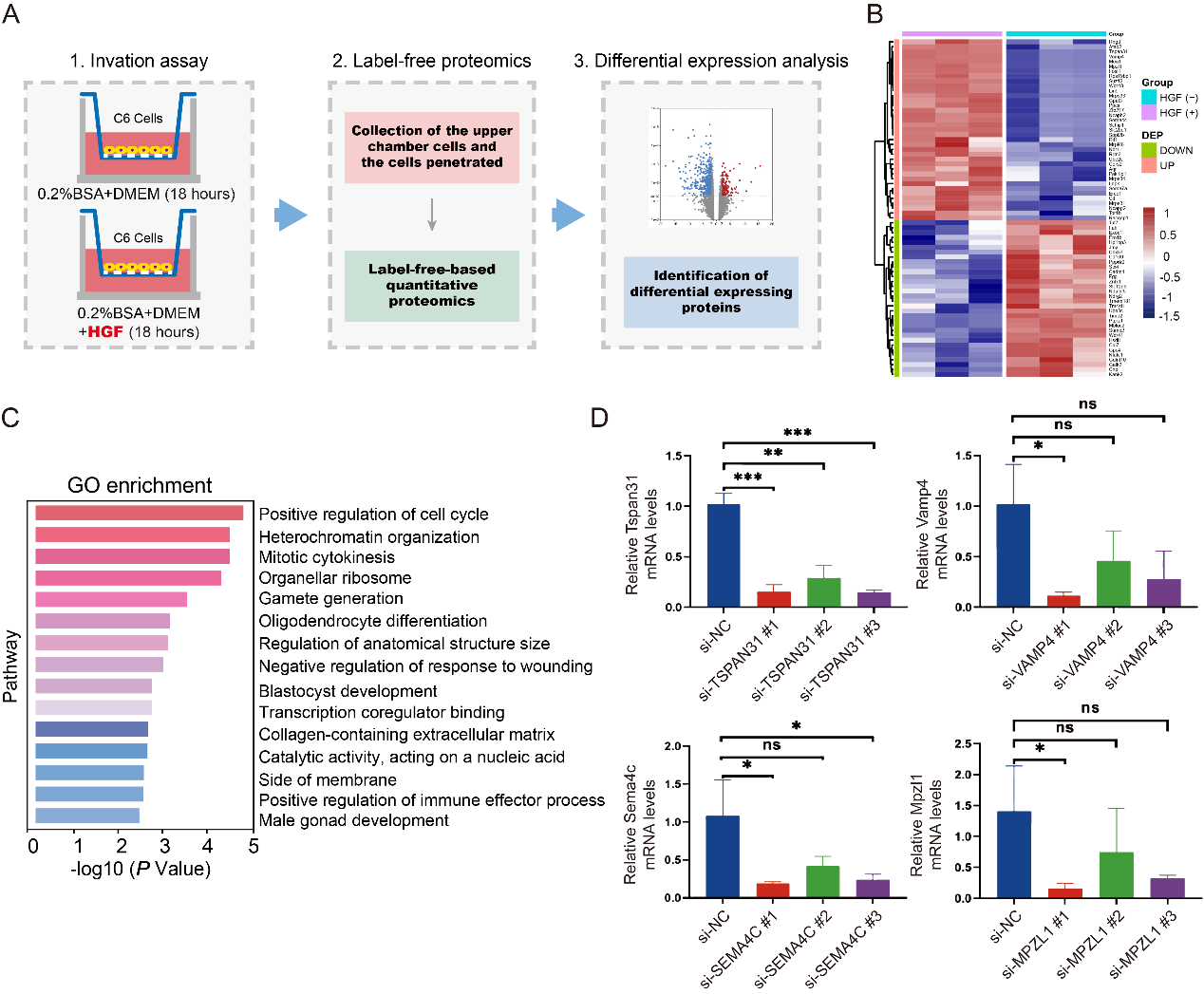
**Figure S2**

**Figure S3**


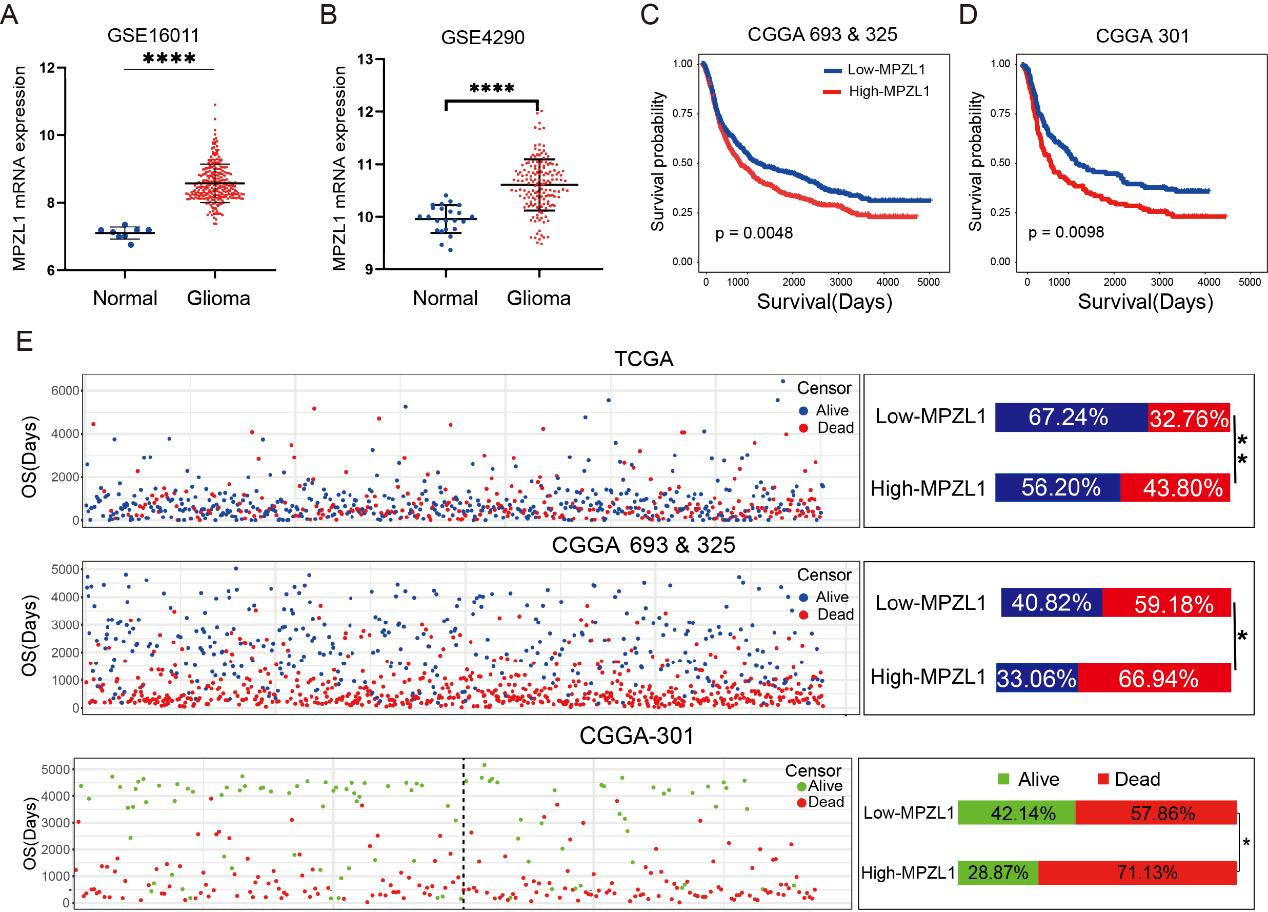


**Figure S4**


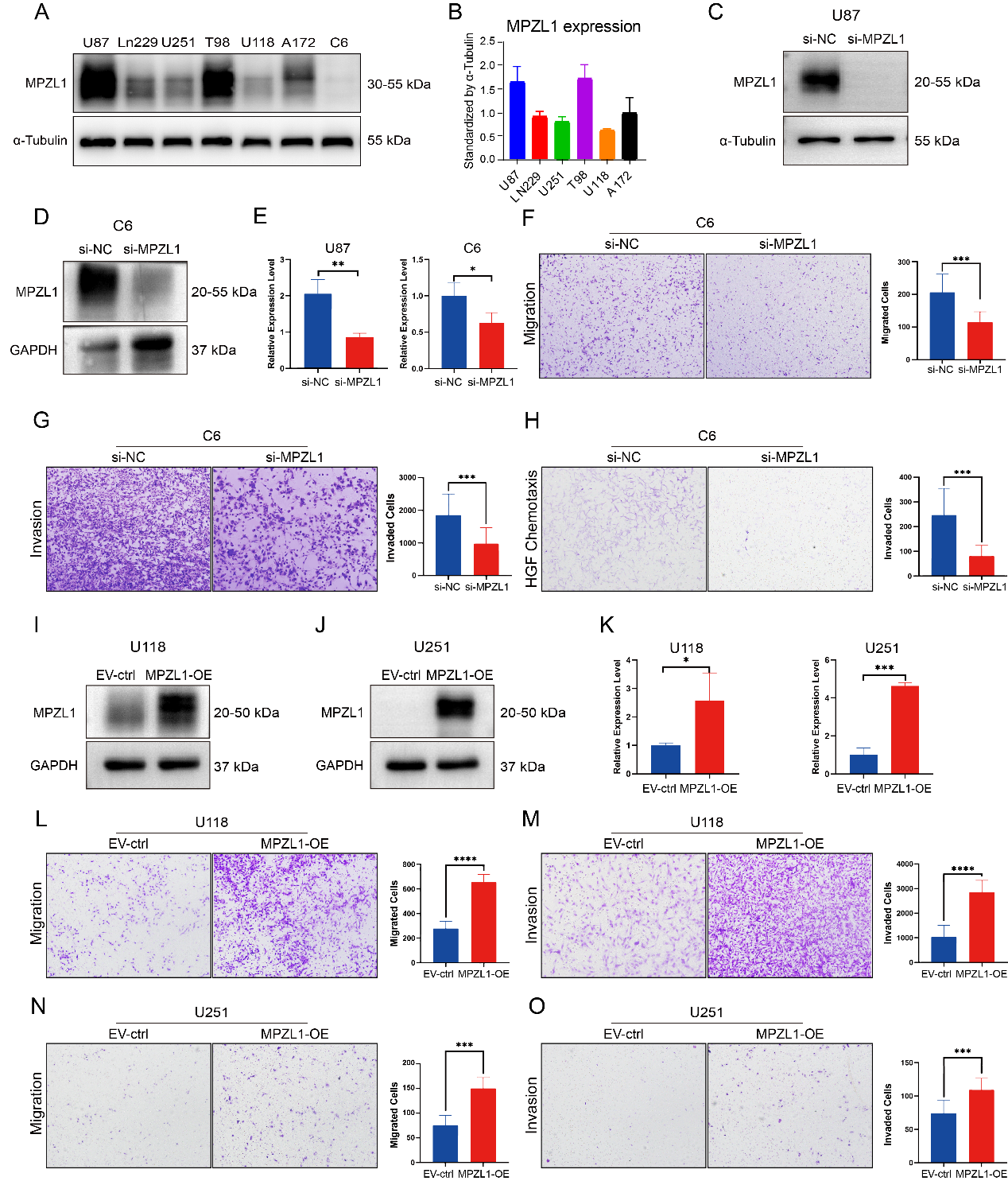


**Figure S5**


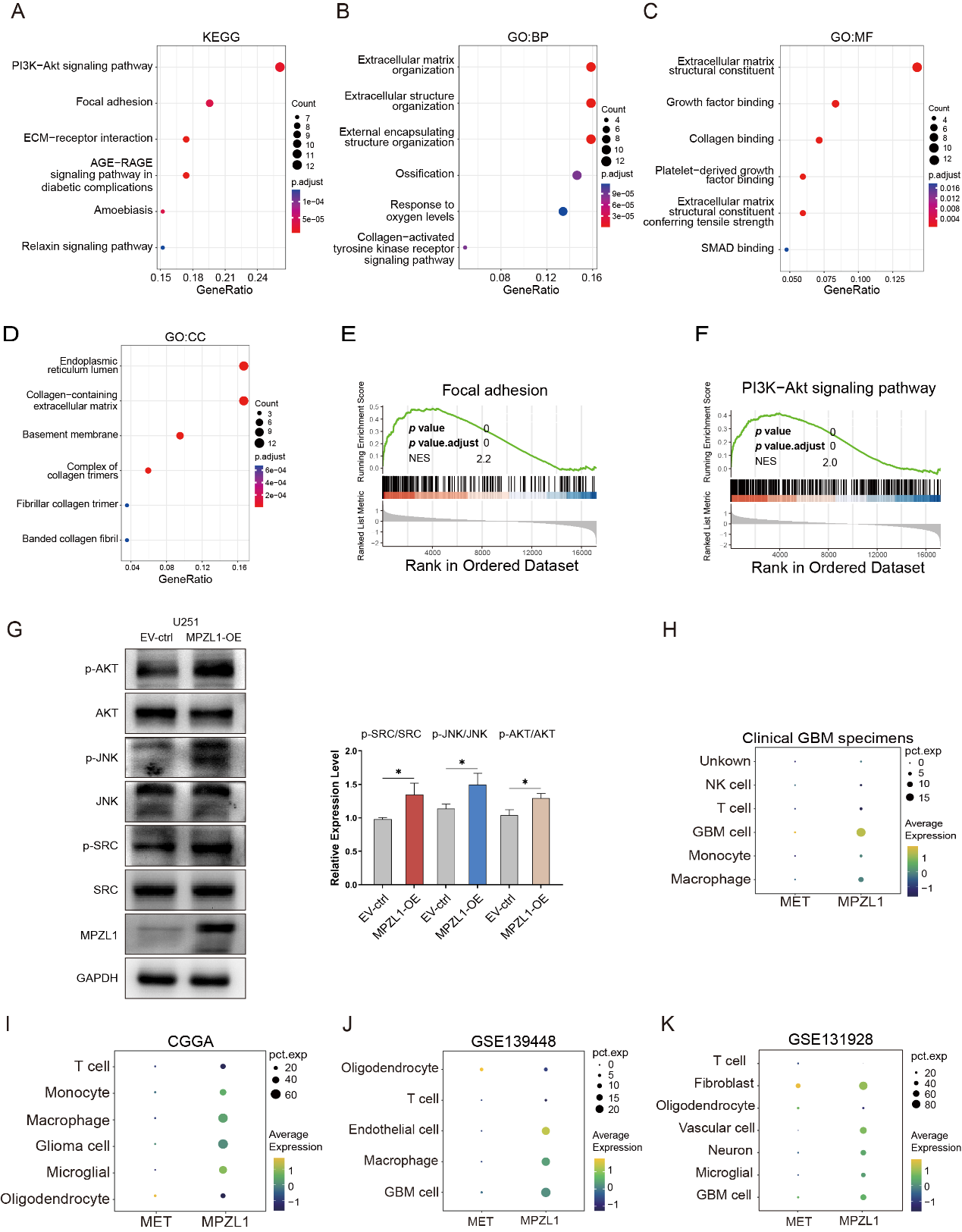


**Figure S6**

**
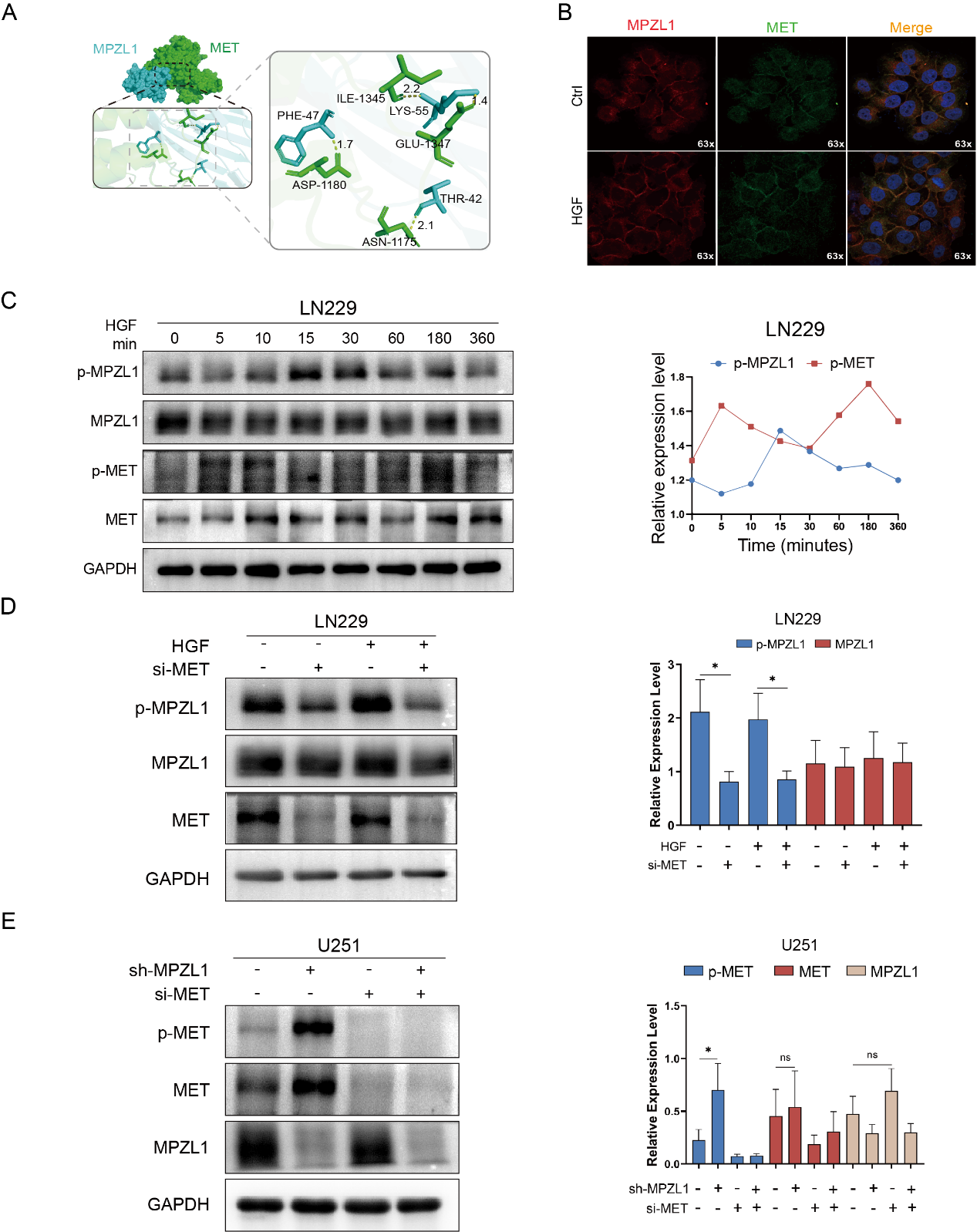
**
