# Supplementary material for: MPZL1 as an HGF/MET signaling amplifier promotes cell migration and invasion in glioblastoma
Source: Genes Dis. 2023 Sep 9;11(5):101085. doi: 10.1016/j.gendis.2023.101085 (PMC11176650; doi:10.1016/j.gendis.2023.101085)
Supplement: Multimedia component 5 [file mmc5.docx]

**Materials and Methods**

Clinical specimens

All glioma samples were obtained after surgical resection from patients admitted to the Department of Neurosurgery, Nanfang Hospital, Southern Medical University, China, and the corresponding clinical data were collected. The glioma specimens were obtained for pathological examination and cell isolation. This study was approved by the Ethics Committee of Nanfang Hospital, Southern Medical University. Written informed consent was provided by all patients. Clinical data of patients were obtained from Nanfang Hospital.

Cell culture

Established Rat GBM cell line C6 and human GBM cell lines U87, LN229, U251 and U118 were purchased from the American Type Culture Collection (ATCC, USA). Cell lines were cultured with DMEM (Thermo Fisher, 11320033, USA) supplemented with 10% fetal bovine serum (Biological Industries,040011ACS, Israel) and penicillin-streptomycin (100 U/mL) (Gibco, 15140122, USA) in humidified incubators at 37℃ with 5% CO2.

Cell transfection and infection

At 70% confluence, cells were transfected with 50μM siRNA using Lipofectamine (Lipo) 2000 (Invitrogen, USA, #11668019) according to the manufacturer' s protocol. Lentiviral transduction was performed according to the manufacturer' s instructions. Quantitative real-time PCR (Q-PCR) and Western blot were used to monitor the transfection efficiency. The siRNA sequences used are listed in **Table S3**. RNA extraction was performed 48 h after transfection for RNA sequencing. Total protein was extracted 48-72 h after transfection.

Reverse transcription polymerase chain reaction (RT-PCR)

Total RNA was extracted from C6 cells using RNA extraction kits (FOREGENE, RE-03111, China), followed by reverse-transcript PCR by using the PrimeScript RT master mix (TaKaRa, RR047A, Japanese), according to the manufacturer' s protocols. Subsequently, the resulting complementary DNA (cDNA) was used as templates for PCR amplification by using the 2 × ChamQ SYBR qPCR Master Mix (Vazyme, Q311-02-AA, China) in a CFX96 Real-Time PCR System (Bio-Rad). For Q-PCR, the standard ΔΔCT method was used to calculate the expression levels of targeted messenger RNAs (mRNAs), using GAPDH as a reference gene. Primers for targeted mRNAs are listed in **Table S3**.

Western blot

Total protein was extracted from GBM cells 96 hours after transfection with siRNAs or infection with lentivirus by RIPA buffer (KeyGEN BioTECH, KGP702, China) supplemented with protease inhibitor cocktail (NCM Biotech, P002, China). Protein samples were quantified by colorimetric bicinchoninic acid assays (NCM Biotech, WB6501, China). The proteins were then denatured by boiling with 1× loading buffer, separated by SDS-PAGE gels, and transferred to a polyvinylidene difluoride (PVDF) membrane (Millipore, ISEQ00010, Ireland). The PVDF membranes were blocked with 5% nonfat dry milk (Cell signaling technology, 9999S, USA) in TBS-Tween (0.1%) for 1 hour at room temperature. After incubation of primary antibodies and horseradish peroxidase-conjugated secondary antibodies, protein bands on PVDF membranes were visualized by a chemiluminescence kit (ABP Biosciences, FP300, USA) in BLT GelView6000Pro.

scRNA-seq data processing and the identification of cells

The CellRanger 2.2.0 (10× Genomics) analysis pipeline was used to generate a digital gene expression matrix from these data according to its guidelines. The raw digital gene expression matrix (UMI counts per gene per cell) was filtered, normalized, and clustered using R 3.5.2 software. Cell and gene filtering was performed as follows. For each detected cell, UMIs were less than the (1-doublet rate) to exclude multiplets. The multiplet rates were determined according to the user guide provided by 10× Genomics. The UMI was larger than the 8th percentile to exclude ambient bias. Mitochondrial RNA was less than the 90th percentile to exclude devitalized cells. scRNA-seq analysis of all our samples were performed by using Seurat and ClusterProfiler packages, following the reference manual^20-22^. Cell type-specific markers and GO enrichment results were combined to classify the identity of each cluster. Gene expression profiling of MET and MPZL1 in glioma based on TCGA and GTEx (the Genotype-Tissue Expression) databases were carried out by using Gene Expression Profiling Interactive Analysis (GEPIA2)^23;24^.

Data availability

The results here are partially based upon data generated by GEO datasets, the TCGA Research Network: https://www.cancer.gov/tcga and the CGGA project. The scRNA-seq data used in this study are available at the Genome Sequence Archive (https://ngdc.cncb.ac.cn/gsa/) with the accession number of CRA002498.

Statistics

The statistical analyses were performed in GraphPad (8.0). Survival analysis of the data from the TCGA GBM cohort was performed in the GEPIA2 platform or R software, using the Kaplan Meier method to calculate the survival curves. Significance cut-off: ns, not significant; *P <0.05; **P < 0.01; ***P < 0.001; and ****P < 0.0001.
